# A comparative study on droplet characteristics and specific charge of ethanol in two small-scale electrospray systems

**DOI:** 10.1038/s41598-019-55223-6

**Published:** 2019-12-11

**Authors:** Yunhua Gan, Zhengwei Jiang, Haige Li, Yanlai Luo, Xiaowen Chen, Yanling Shi, Yuying Yan, Yunfei Yan

**Affiliations:** 1 School of Electric Power, South China University of Technology, Guangzhou, 510640 China; 2 Teaching and Training Center for Engineering Basis, South China Agricultural University, Guangzhou, 510640 China; 3 Fluids & Thermal Engineering Group, Faculty of Engineering, The University of Nottingham, University Park, Nottingham, NG7 2RD UK; 4 Key Laboratory of Low-grade Energy Utilization Technologies and Systems (Chongqing University), Ministry of Education of China, Chongqing University, Chongqing, 400044 China

**Keywords:** Mechanical engineering, Fluid dynamics

## Abstract

An investigation on the droplet characteristics of ethanol in small-scale combustors with two different systems was conducted experimentally and theoretically. The classical capillary-mesh electrode arrangement was applied in Type A electrospray system, and for Type B, an additional ring electrode is included. The droplet size and velocity were measured by a Phase Doppler Anemometer. The electric filed intensity was theoretically calculated in the two electrospray systems. Compared with Type A, Type B system has smaller droplet size and velocity in the same spraying mode. Meanwhile the electrospray process in Type B system is more stable than that in Type A with its smaller root mean square velocity. By measuring the spraying current, the average specific charge of the droplets for the two systems was obtained in different spraying modes. And it was found that the addition of the ring electrode can help to increase the droplet charge, which is the fundamental reason for Type B electrospray system to perform better. The corona charge of the droplets was theoretically calculated for the two electrospray systems. It was found that the calculated specific charge generated by corona charging was in good agreement with the experimental results.

## Introduction

The electrospray technique has many irreplaceable advantages. For example, it can yield quasi-monodisperse droplets, control the droplet motion easily and avoid coalescence of droplets^1^. It has been applied in many areas such as fuel atomization in combustion systems^2,3^, controlled droplet generation^4,5^, electrospinning^6,7^, thin film deposition^8,9^ and electrospray ionization-mass spectrometry applications^10,11^. Meanwhile more and more attention has been focused on the micro-power systems using micro-scale combustors due to the higher specific energy of liquid hydrocarbons than that of batteries^12,13^. But it is difficult to keep stable flame inside the micro-combustor, due to limitation by short residence time and high heat loss rate^14,15^. Some researchers have applied the electrospray to atomize the fuel in micro-combustor.

Electrospray technique is proved to be very suitable in micro/meso-combustors and can improve the combustion performance and stability. Based on the electrospray, Kyritsis *et al*.^3^ studied the combustion performance of the heavy liquid hydrocarbon JP8 in a mesoscale combustor, and the catalyst formulation was coupled to achieve higher efficiency and lower CO emission. Deng *et al*.^16^ proposed an improved combustor design and extended to the multiplexed electrosprays. The microcombustor can achieve a very high release rate (270 MW/m^3^). Yuliati *et al*.^17^ successively established a stable flame inside a narrow tube with the inner diameter of 3.5 mm, the electrospray technique was employed to atomize the liquid fuel. Mikami *et al*.^18^ extended the work of Yuliati *et al*. and studied the flame stability. Gan *et al*.^19^ studied the combustion characteristics with the two different combustors which employed different electrode structures, i.e., capillary-mesh and capillary-ring-mesh respectively, and it is found that the capillary-ring-mesh combustor yielded better performance in the combustion efficiency and emissions. The electrospray characteristics are changed when the ring electrode is added in system and then the combustion characteristics are affected. It is necessary to study the effect of ring electrode on electrospray.

Meanwhile many researchers focus on the electrospray characteristics in the classical capillary-mesh system. Jaworek and Krupa^20^ classified the electrospray modes by using the multi capillary-mesh systems where the capillary was maintained at high electric potential and the plate was connected to the ground. Deng and Gomez^21^ studied the complete transient response of Taylor cones subject to a step change in external electric field with capillary-mesh system. Morad *et al*.^22^ improved the stability of the Taylor cone-jet and achieve a very high throughput by installing a hemispherical cap above the capillary. Recently, the electrospray in capillary-ring-mesh system have also been studied. Xie and Wang^23^ performed electrospray in the dripping mode to realize droplet formation and found that the addition of a ring electrode can help to stabilize the process. Zhao *et al*.^24^ found that for a typical three-electrode arrangement (capillary-ring-mesh system) of electrospray used in practical application, a strong charging field can be established with a relatively low voltage. Chen *et al*.^25^ developed a new electrospray design by placing a ring electrode behind the capillary tip, and they found that the electric field strength could be enhanced. Most of studies are about the characteristics at cone-jet mode because the key of electrospray is the Taylor cone^26,27^. Despite of those researches, a clear and detailed picture of the ring electrode on the electrospray is still lacking. Especially, there remains an unknown area in the addition of the ring electrode on the droplet size, velocity and specific charge during the electrospray process.

The present study is aimed to investigate the difference of electrospray characteristics between two small-scale electrospray systems. The effects of adding an extra ring electrode on the electrospray are the focuses in the present study. With these in mind, a series of comparative experiments were conducted to explore the effects of a ring electrode on the electrospray performance, including the droplet size, droplet velocity and specific charge. Moreover, the electric field of the two different electrode systems was obtained to reveal the role of the ring electrode. Last, the corona charge of the droplets was calculated and compared with the experimental results with the attempt to theoretically estimate the droplet charge. The results may provide some help for the design of a micro-scale combustion system based on electrospray technique reasonably.

## Experimental Methods

### Experimental setup

**Figure 1:**
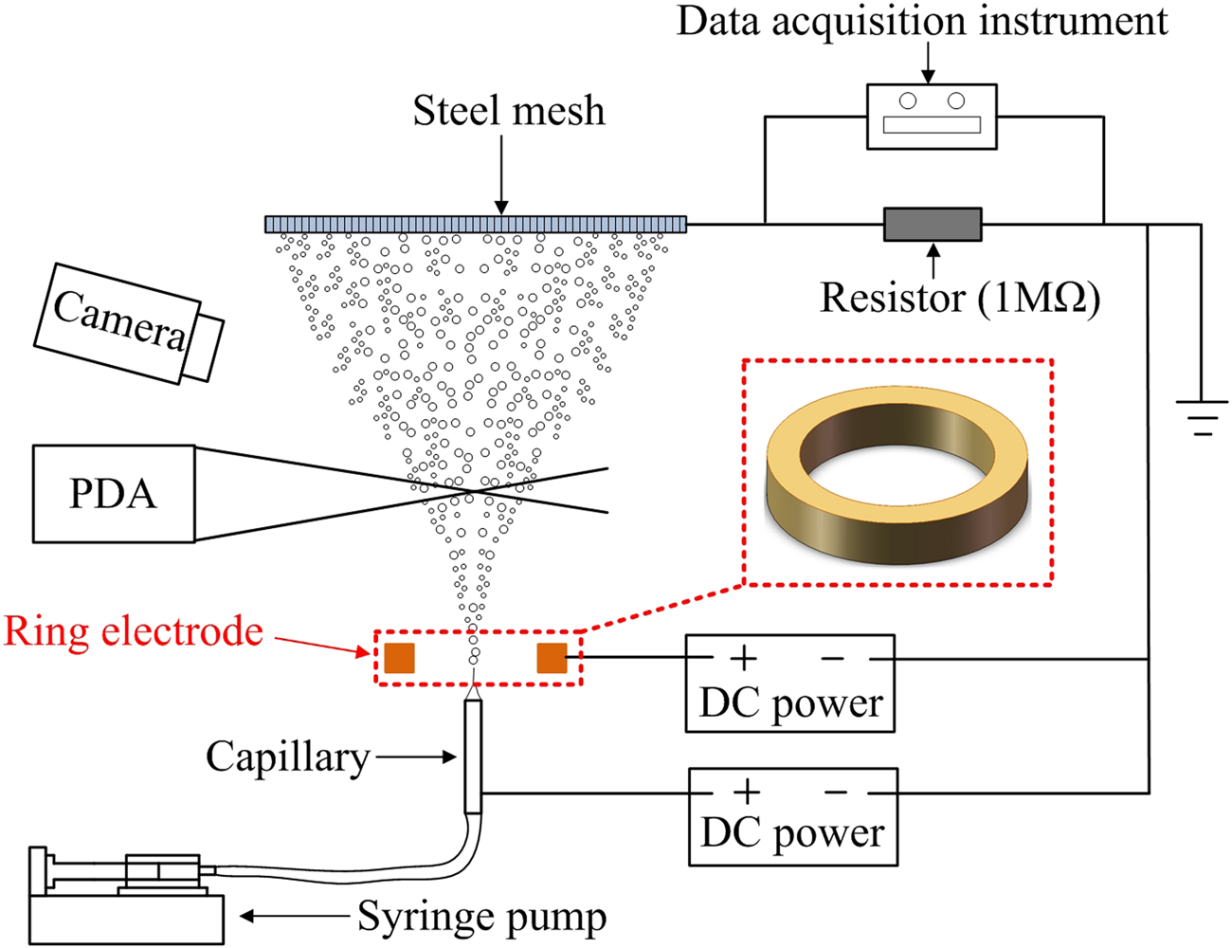
Schematic of the experimental setup for two electrospray systems. Type A system contains no ring electrode, and Type B system contains a ring electrode which is placed above the capillary.

The schematic of the experimental setups for two electrospray systems are presented in Fig. 1. The two systems are the same except for the ring electrode. The system contains no ring electrode is defined as Type A, and the one contains a ring electrode which is placed above the capillary is defined as Type B. Ethanol was supplied into the capillary by a syringe pump (KDS100, KD Scientific, USA) and the volume flow rate was kept at 1.0 ml/h. The capillary and the ring electrode were connected to the positive electrode of two DC power sources (71020 P, GENVOLT, UK) respectively. The mesh was connected to the ground electrode of a DC power source. The electric current in the electrospray process was determined by having a resistor (1 MΩ) in a series circuit between the mesh and the ground. The voltage drop across the resistor was measured using a data acquisition instrument (34970 A, Agilent, USA), and the electric current was calculated using Ohm’s law. The size, velocity, root mean square (RMS) velocity of droplets was measured by a Phase Doppler Anemometer (PDA) (classic PDA, Dantec Dynamics, DK). The spraying modes were visualized by a digital camera (EOS 5D Mark III, Cannon, Japan). Experiments were carried out in ambient temperature. Physical properties of ethanol at 298 K are shown in Table 1.

**Table 1:** Physical properties of ethanol (298 K).

Properties	Values
Surface tension (N/m)	0.022
Dynamic viscosity (mPas)	1.16
Density (kg/m^3^)	789
Relative permittivity	25.3
Electrical conductivity (S/m)	5.1 × 10^−5^
Purity (%)	≥99.7

### Test section

**Figure 2:**
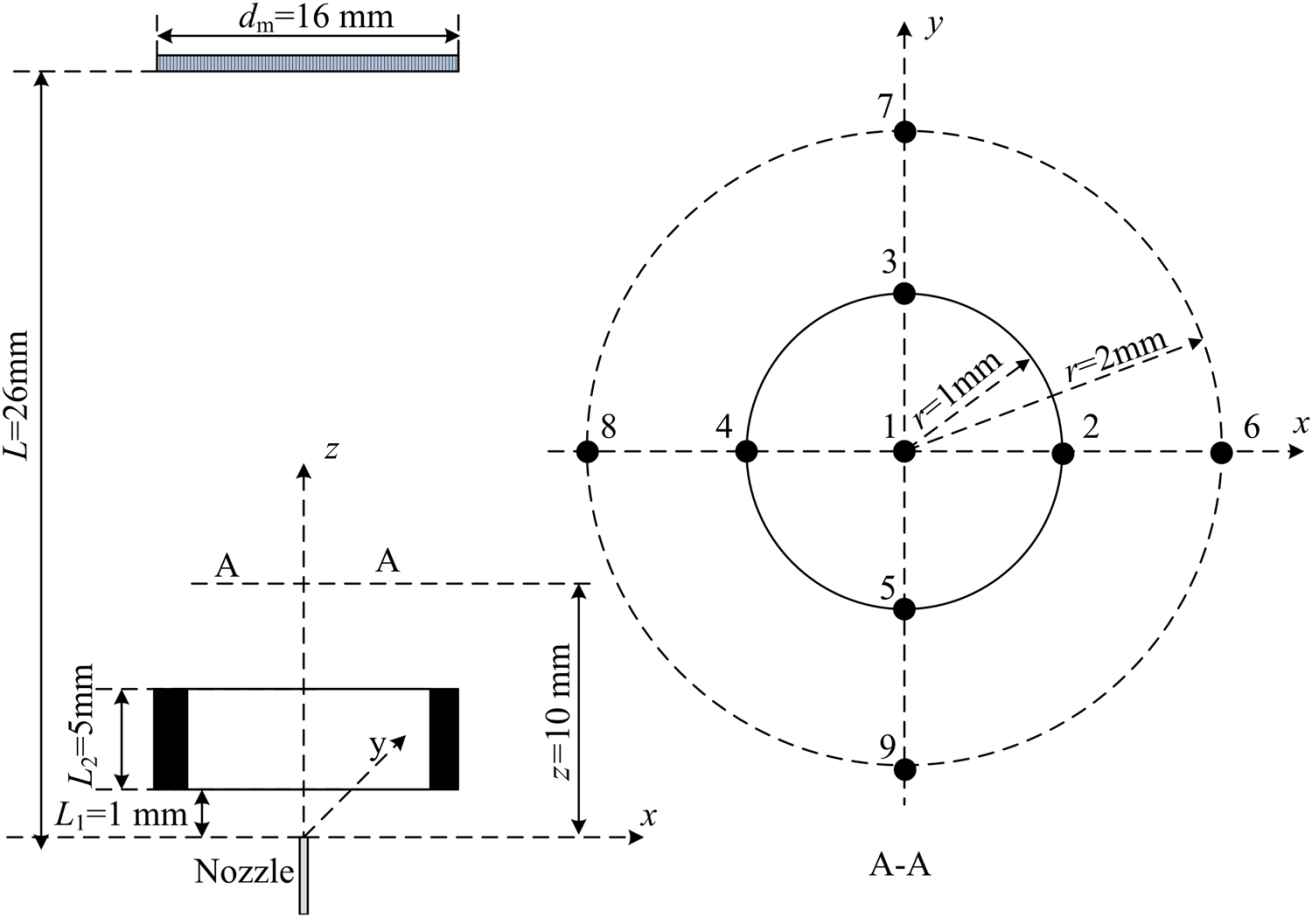
The schematic of measurement points distribution.

The test section of the present experiment and distribution of measurement points are shown in Fig. 2. The voltage on capillary electrode is *V*, and voltage on ring electrode is *V*_*r*_. The inner diameter and outer diameter of the capillary electrode are *d*_inner_ = 0.9 mm and *d*_outer_ = 1.2 mm respectively. The inner diameter and outer diameter of the ring electrode are *D*_inner_ = 12 mm and *D*_outer_ = 16 mm, respectively, and the height *L*_2_ = 5 mm. The steel mesh has a diameter of 16 mm. *L* = 26 mm is the distance between capillary outlet and steel mesh and *L*_1_ = 1 mm is the distance between capillary outlet and the lower surface of the ring electrode.

The measurements section are performed at *z* = 10 mm above the tip of the capillary. Measurement points distribute on the concentric circles with different radiuses (*r* = 1 mm and *r* = 2 mm).

### Specific charge measurement

The specific charge is calculated as^19^,

1 $$\beta =\frac{q}{m}=\frac{It}{{Q}_{m}t}=\frac{{V}_{0}/{R}_{0}}{{Q}_{v}\rho }$$

where *β* is the specific charge (C/kg), *q* is the charge of droplet (C), *m* is the mass of droplet (kg), *Q*_*m*_ is the mass flow rate (kg/s), *Q*_*v*_ is the volume flow rate (m^3^/s), *ρ* is the density of ethanol (kg/m^3^), *R*_0_ is the resistance (1 MΩ), *V*_0_ is the voltage (V) on the resistance. *V*_0_ is measured by data acquisition instrument, and *Q*_*v*_ is controlled by a syringe pump.

### Error analysis

In the experiments, the direct measurement error comes from the uncertainties of the measuring tools or systems, such as data acquisition instrument and PDA. In another case, some parameters (specific charge in this study) cannot be measured directly and are calculated based on other direct measured parameters, which brings the indirect measurement error^28^.

2 $${\sigma }_{y}=\sqrt{\mathop{\sum }\limits_{{\rm{j}}=1}^{{\rm{n}}}{(\frac{df({x}_{1},{x}_{2},\ldots ,{x}_{{\rm{j}}})}{d{x}_{{\rm{j}}}})}^{2}{\sigma }_{{\rm{xj}}}^{2}}$$

where *x*_j_ is directly measured parameter, *σ*_xj_ is the error of directly measured parameter, and *σ*_y_ is the error of indirectly measured parameter. The important errors are summarized in Table 2.

**Table 2:** Error analysis.

Parameters	Measurement tool	Ranges	Error
Flow rate, *Q*_*v*_	Syringe pump	1.0 ml/h	±1.0%
Voltage, *V*	DC power supply	0–6.86 kV	±1.0%
Voltage on the resistance, *V*_0_	Data acquisition instrument	0–1 mV	±1.0%
Specific charge, *β*	—	0–2 C/kg	±1.7%
Diameter, *D*_32_	Particle Dynamics Analysis	0–250 μm	±1.0%
Axial velocity, *v*	Particle Dynamics Analysis	0–340 m/s	±1.0%
Diameter, *d*_inner_, *d*_outer_, D_inner_, D_outer_	Vernier caliper	0.9–1.2 mm	±0.02 mm
Length, *L*, *L*_1_, *L*_3_	Vernier caliper	16–80 mm	±0.02 mm

## Results and Discussion

### Electric field distribution

Jones and Thong^29^ investigated the electric field configuration between the capillary and the ground electrode. The calculated electric potential and electric field intensity were:

3 $$\varPhi =\frac{-q}{4\pi {\varepsilon }_{0}}\,\mathrm{ln}(\frac{{({r}^{2}+{z}^{2})}^{1/2}+z}{(2{z}_{0}-z)+{({r}^{2}+(2{z}_{0}-z))}^{1/2}})$$

4 $$E=\frac{-q}{4\pi {\varepsilon }_{0}}\,\mathrm{ln}(\frac{1}{{\{{r}^{2}+{(2{z}_{0}-z)}^{2}\}}^{1/2}}+\frac{1}{{({r}^{2}+{z}^{2})}^{1/2}})$$

where *Φ* is the electric potential, *E* is the electric field intensity, *q* is the droplet charge. The coordinate of electrode system is shown in Fig. 1. *z*_o_ is the distance between the capillary outlet and steel mesh, *r* (radial direction) is the distance from the center of capillary outlet to right and *z* (axial direction) is the distance from the center of capillary outlet vertical to the steel mesh. When *r* = *r*_*c*_ and *z* = 0, *Φ*_*0*_ = *V. r*_c_ is the radius of capillary and it is supposed to the mean value of inner and outer radius of capillary and *r*_c_=0.525 mm. *V* is the voltage applied to the capillary. Based on those data, the Eqs. (5) and (6) can be derived as,

5 $${\varPhi }_{0}=\frac{q}{4\pi {\varepsilon }_{0}}\,\mathrm{ln}(\frac{{({{r}_{c}}^{2}+4{{z}_{0}}^{2})}^{1/2}+2{z}_{0}}{{r}_{c}})$$

6 $$q\approx \frac{4\pi {\varepsilon }_{0}{\varPhi }_{0}}{\mathrm{ln}(4{z}_{0}/{r}_{c})}$$

The system of Type A is similar to Jones’s assumption, so the axial electric field in Type A, *E*_*A*_, is calculated by Eq. (4). When *r* = 0, the formula is shown in Eq. (7). According the derivation of Shi *et al*.^30^ and Ru *et al*.^31^, the axial electric field of the single ring electrode-grounded steel mesh system, *E*_r_, is shown in Eq. (8). The ring electrode charge density, *η*, is shown in Eq. (9)

7 $${E}_{A}=\frac{q}{4\pi {\varepsilon }_{0}}\,\mathrm{ln}(\frac{1}{2{z}_{0}-z}+\frac{1}{z})$$

8 $${E}_{r}=\frac{\pm \eta {D}_{outer}(z-{L}_{3})}{2{\varepsilon }_{0}{({{D}_{outer}}^{2}+{(z-{L}_{3})}^{2})}^{3/2}}-\frac{\pm \eta {D}_{outer}(2{z}_{0}-{L}_{3}-z)}{2{\varepsilon }_{0}{({{D}_{outer}}^{2}+{(2{z}_{0}-{L}_{3}-z)}^{2})}^{3/2}}$$

9 $$\eta =\pi {\varepsilon }_{0}{V}_{r}$$

where *D*_*outer*_ is the radius of ring electrode and *D*_*outer*_ = 8 mm, *V*_*r*_ is the voltage applied to the ring electrode.

According to the superposition principle of electrostatic field, the axial electric field in Type B, *E*_*B*_, is composed by the axial electric field of the single capillary electrode-grounded steel mesh system (Type A system) and the single ring electrode-grounded steel mesh system. Its formula is shown in Eq. (10).

10 $${E}_{B}={E}_{A}+{E}_{r}$$

**Figure 3:**
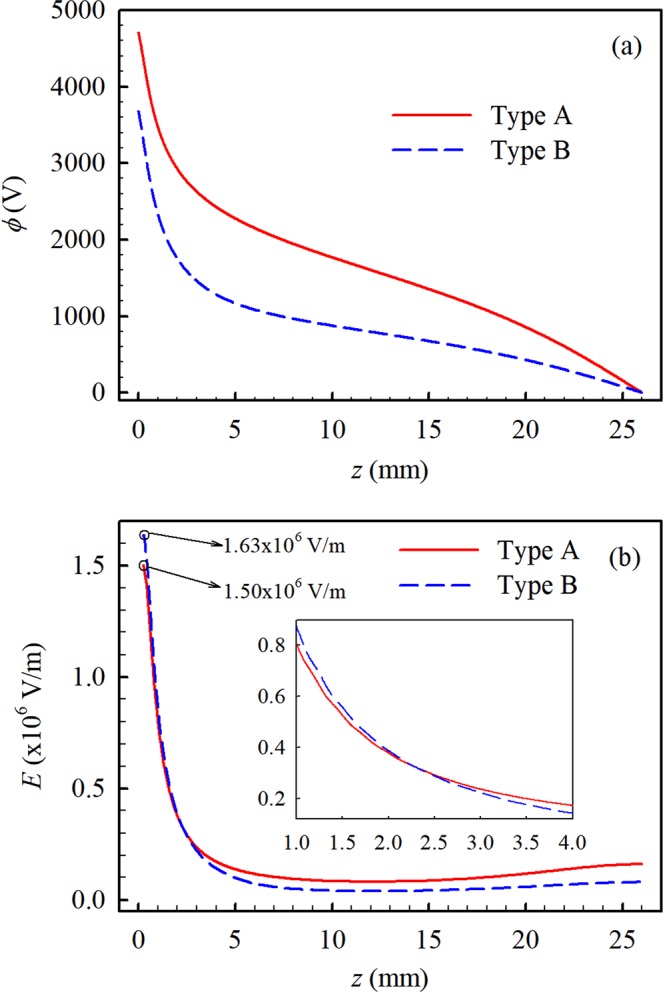
The distributions of axial potential **(a)** and electric field **(b)** in two electrospray systems (Type A: *V* = 5 kV; Type B: *V* = 4 kV, *V*_*r*_=1 kV).

To perform an objective comparison, the axial potential and electric field in two electrospray systems are calculated with their total applied voltage being the same, that is, for Type A, *V* = 5 kV and for Type B, *V* = 4 kV, *V*_*r*_ = 1 kV, and the results are shown in Fig. 3. It is shown in Fig. 3(b) that electric field intensity in two electrospray systems decreases rapidly away from the capillary outlet. And it is noted that for Type B, the electric field between the capillary and ring electrode is stronger. Above the ring electrode, the trend is reversed and the electric field for Type B is weaker. It is evidently since there is only a 1 kV voltage drop within a large distance, from the ring to the mesh.

### Droplet size

The droplet size *D*_32_ was measured by PDA at the flow rate *Q*_v_ = 1.0 ml/h. *D*_32_ can be defined as follows:

11 $${D}_{32}=\frac{{\sum }_{i=1}^{N}\,{D}_{{\rm{i}}}^{3}}{{\sum }_{i=1}^{N}\,{D}_{{\rm{i}}}^{2}}$$

where *N* is the total number of droplets (*N* ≤ 2000), *D*_i_ is the diameter of the individual droplets at the specific measuring position. *D*_32_ is known as Sauter Mean Diameter (SMD)^12^. It is well known that droplet size is the most relevant parameter to evaluate the performance of the spraying.

**Figure 4:**
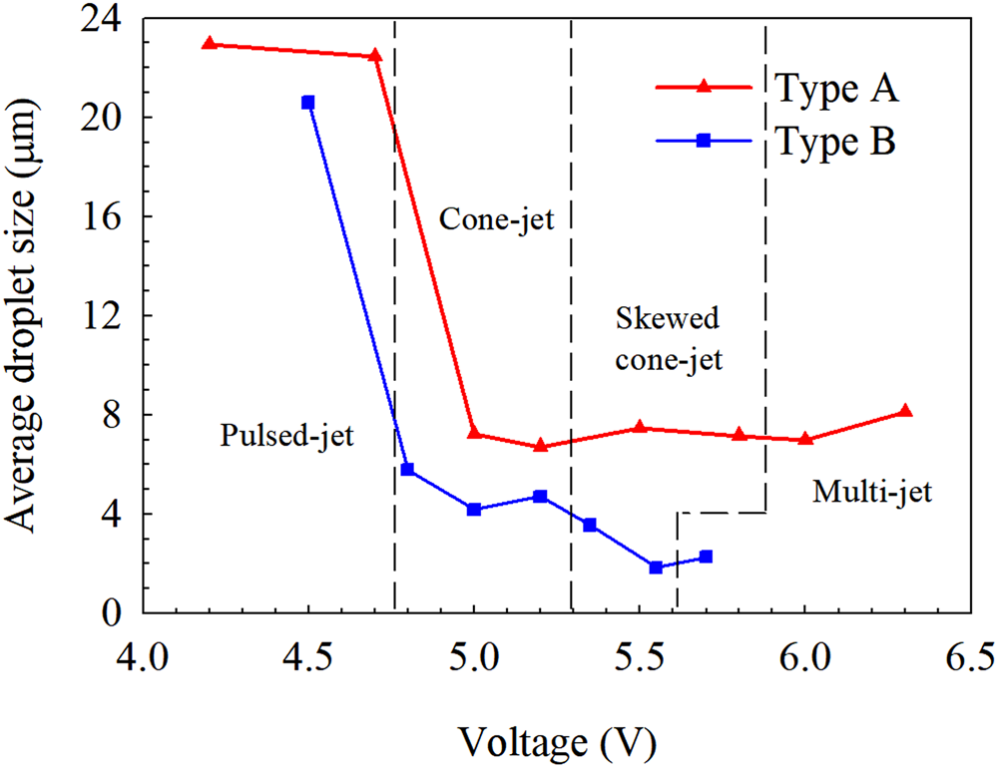
The average droplet size in two electro-electrospray systems.

Four typical spraying modes were observed in Type A and Type B electrospray system when applied voltage changed. The four typical spraying modes are called pulsed-jet mode, cone-jet mode, skewed cone-jet mode and multi-jet mode, which have been described in our previous study^19^. Meanwhile the same spraying mode shows similar characteristics in Type A and Type B systems. Since Type B employs a ring electrode applied with 1 kV voltage, the required capillary voltage to achieve the same spraying mode (the same level of spraying performance) is lower than that of Type A, as can be seen in Fig. 4. Thus, it is inappropriate to compare the differences between Type A and B at the same capillary voltage. In this work, we conducted the comparison at the same total applied voltage (for Type A, it is the capillary voltage and for Type B, it equals to the sum of the capillary voltage and ring voltage). Besides, the regimes of different spraying modes were indicated.

The droplet size distribution at cross-section of z = 10 mm in two electrospray systems is given in Fig. 4. The droplet size at pulsed-jet mode is the largest both in two electrospray systems. At the pulsed-jet mode, the electrospray presents the oscillation characteristic^19^. This will result in a relatively thick jet which then breakups into large droplets. Comparing the droplet size distribution in two electrospray systems in Fig. 4, it is found that the droplet size in Type B is smaller than that in Type A in the same spraying mode. As shown in Fig. 3(b), the electric field near the capillary (z < 2 mm) of Type B is stronger than that of Type A. Since the process of the jet breaking up into droplet mainly occurs in the vicinity of the capillary (z < 2 mm), under the effect of larger electrostatic force, Type B can produce droplets with smaller size.

### Droplet velocity

**Figure 5:**
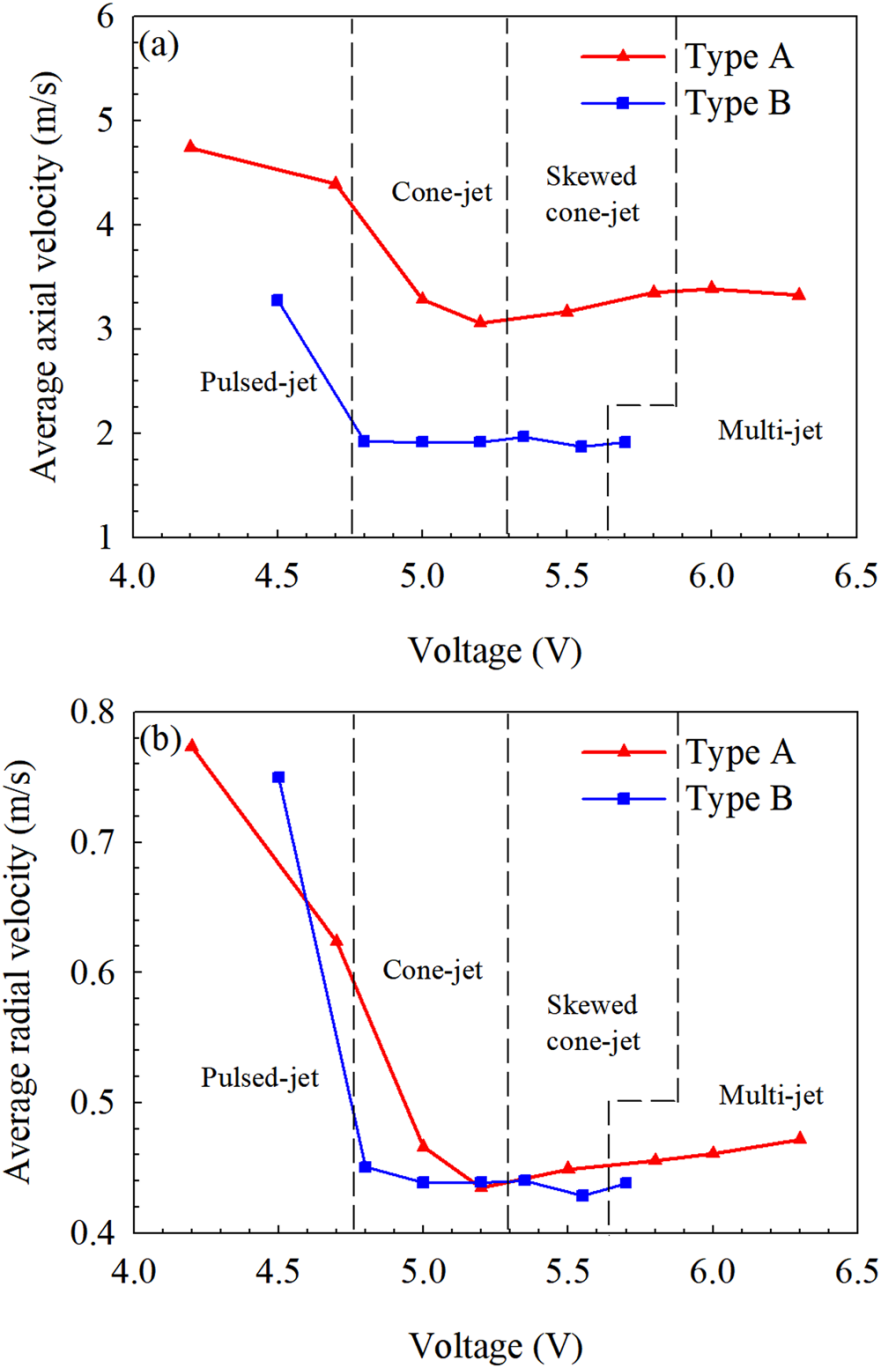
The average axial (**a**) and radial (**b**) velocity in two electrospray systems.

Figure 5 shows the distribution of axial and radial velocity at four spraying modes in Type A and Type B systems. Comparing the axial velocity distribution in two electrospray systems in Fig. 5(a), it is found that the axial velocity in Type B is lower than that in Type A in the same spraying mode. Especially, the axial velocity of Type B is close to 2 m/s and that in Type A is about 3 m/s at the cone-jet mode, skewed cone-jet mode and multi-jet mode. The radial velocity of the two systems is given in Fig. 5(b), and it shows that the difference between the two systems is small. From Fig. 3(b), it is seen that the electric field in Type A is stronger than that in Type B at the cross-section of z = 10 mm, where the PDA measurements is carried out. Since the droplets in the space are driven by the electric field, the stronger electric field in Type A leads to the greater droplet velocity. Furthermore, it also can be seen that the droplet velocity at the pulsed-jet mode is larger than that at others modes. This is mainly due to the difference in the spraying mechanism. At the pulsed-jet mode, the electric field is weak and the electrostatic force acting on the liquid can hardly overcome the surface tension, so the emission of the droplets resulting from the jet breakup takes place discontinuously. In this situation, the velocity of the droplets is relatively large due to the pulse effect.

At other spraying modes (cone-jet, skewed-jet and multi-jet), for Type A, the droplet velocity increases with the voltage and the corresponding modes due to the increasing electric field. However, for Type B, the droplet velocity at these modes is almost the same, which can be contributed to the shielding effect of the ring electrode since the voltage on the ring is kept unchanged. With the presence of the ring electrode, the electric field between the ring and mesh is almost independent with the voltage on the capillary.

The RMS velocity was derived from the PDA measured velocity profiles using the following equation^12^:

12 $${v}_{{\rm{RMS}}}=\sqrt{\mathop{\sum }\limits_{{\rm{i}}=1}^{N}\,{({v}_{{\rm{mean}}}-{\nu }_{{\rm{i}}})}^{2}/N}$$

where ${v}_{{\rm{RMS}}}$ represents standard deviation of the axial velocity of the droplets, ${v}_{{\rm{mean}}}$ is mean velocity at the specific measurement point, *v*_i_ is the velocity of individual droplet, *N* is the total number of droplets at the specific measurement point (*N* ≤ 2000). It is well known that the lower RMS velocity indicates the more stable spraying process.

**Figure 6:**
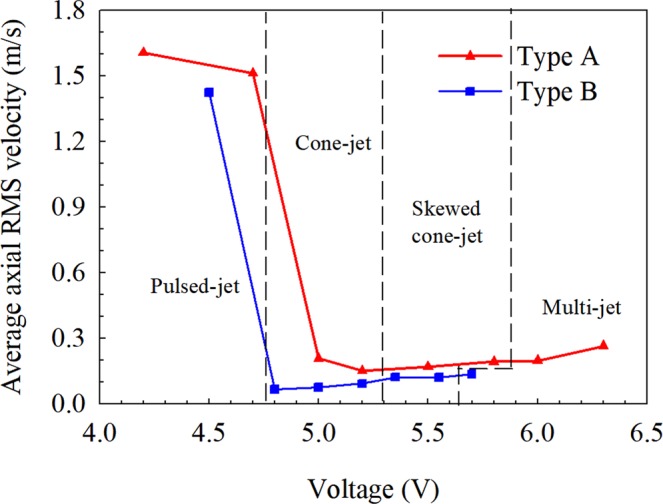
The average axial RMS velocity in two electrospray systems.

Figure 6 shows the axial RMS velocity of four spraying modes in the Type A and Type B systems. It is obviously found that RMS velocity of Type A and Type B at pulsed-jet is larger than that at other spraying modes. It means that the velocity fluctuates visibly at pulsed-jet but fluctuates slightly at other spraying modes.

By comparing the axial RMS velocity distributions of two electrospray systems in Fig. 6, it is found that axial RMS velocity of Type A is close to 0.3 m/s at cone-jet mode, but that of Type B is close to 0.1 m/s. In other words, axial RMS velocity of Type B is smaller than that of Type A. It means that ring electrode plays an important role in the stability of spraying process.

### Specific charge

Three methods usually are used for charging droplets which include corona charging, conduction charging and induction charging. Corona charging means that the droplets are charged by ionic bombardment and it can be used for both conductive liquid and non-conductive liquid. The difference of conduction and induction charging is that in conduction charging the power supply directly contacts the liquid and in induction charging the power supply is connected to an induction electrode^32^. The total droplet charge of electrospray is composed of corona charging, conduction charging and induction charging^33,34^. The relation among the total droplets specific charge *β*_total_ and other specific charge is:

13 $${\beta }_{{\rm{total}}}={\beta }_{1}+{\beta }_{2}+{\beta }_{3}$$

where *β*_1_, *β*_2_, *β*_3_ respectively mean specific charge generated by corona charging, specific charge generated by conduction charging, and specific charge generated by induction charging.

**Figure 7:**
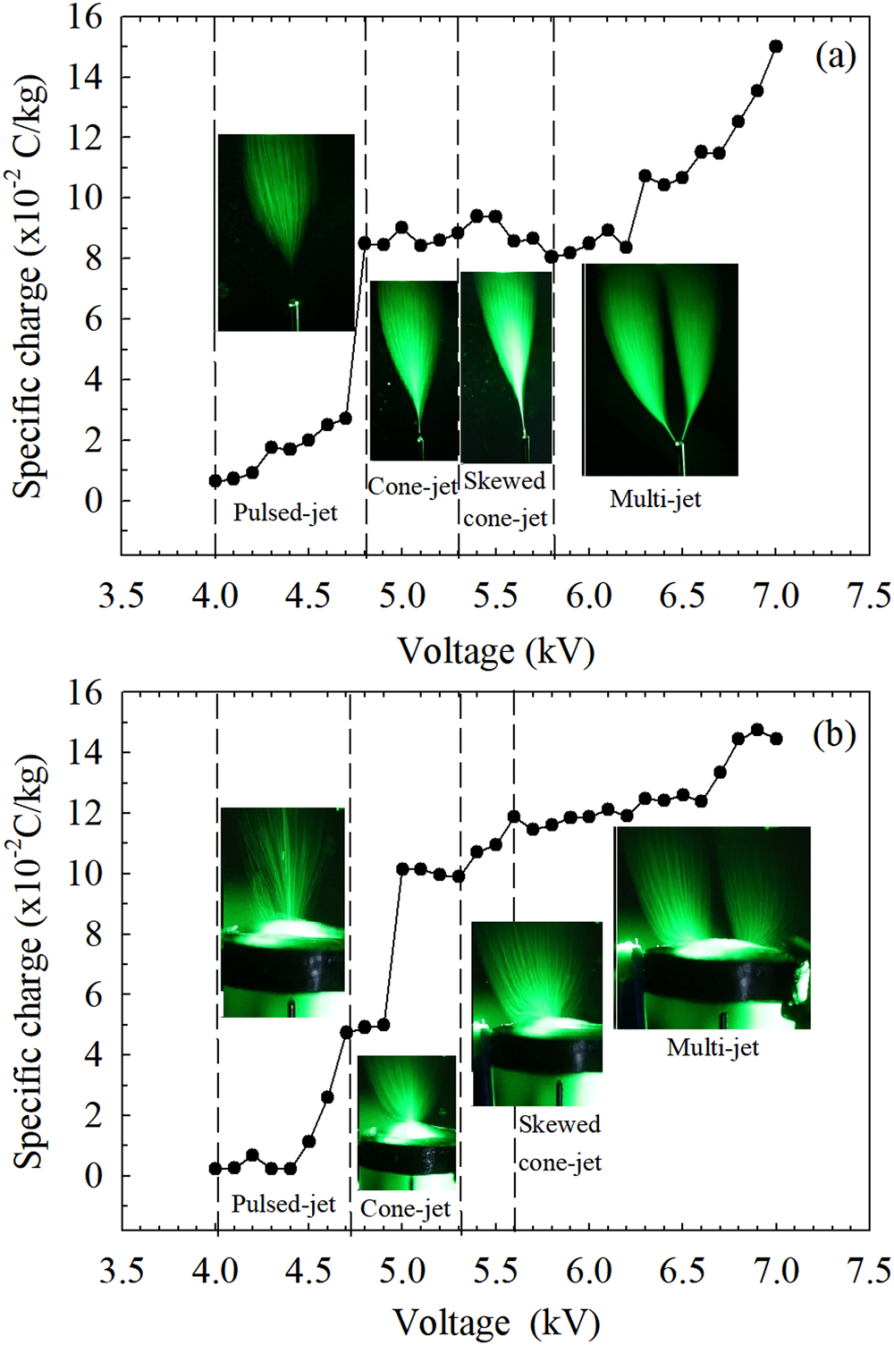
The relationship among total applied voltage on capillary and specific charge and spraying modes in two electrospray systems. **(a)** Type A: capillary-mesh system; **(b)** Type B: capillary-ring-mesh system (*V*_*r*_ = 1 kV).

The relationships among capillary applied voltage, specific charge and spraying modes in two electrospray systems are shown in Fig. 7. It shows that the specific charge increases with increasing of the applied voltage on capillary with two electrospray systems. It is found that specific charge of Type A is different from that of Type B in the same spraying mode. The reason of this phenomenon is that ring electrode will influence the value of specific charge at Type B. But the difference of specific charge between Type A and Type B are small on the whole. It means that ring electrode has little effect on specific charge of droplet, which is consistent with the theory that the electrode geometry has little effect on the spray current^35,36^. In other words, the induction charge generated by ring electrode is small. Because when ethanol is used as spraying liquid droplet and its conductivity is small that it can be regarded as semi-conductive liquid. Simultaneously, the capillary voltage in Type B is lower than that in Type A in the same spraying mode. The reason for this phenomenon is that the induction force is produced by ring electrode around the capillary and it makes ethanol droplet to break up at lower capillary voltage. Though ring electrode has little effect on droplet charge, but electric force on droplet generated by ring electrode still exist.

In addition, the specific charge at the pulsed-jet mode is much smaller than that at other modes. As mentioned above, at the pulsed-jet mode, the electrospray oscillates and the droplets are produced intermittently. Besides, the voltage at this mode is relatively low compared with other modes. The generation rate of charge on liquid is low and consequently, the droplet charge is much smaller. At other spraying modes, a stable meniscus and spraying are established with small disturbances. Therefore, the charged droplets collected by the steel mesh at these cone-jet modes are much more than that at the pulsed-jet mode, which results in the significant increase of specific charge. From Fig. 7(a), it also can be seen at multi-jet mode, the specific charge increases significantly with voltage in Type A. This can be contributed to the dominate effect of the corona charge at this mode. However, for Type B, the influence of the corona charge is partially weakened by the ring electrode so that the increasing trend of the specific charge is not that significant as that in Type A.

The value of the corona charge, conduction charge and induction charge are difficult to be calculated^37^. But the maximum theoretical value of corona charging can be calculated. When an isolated droplet is assumed in the uniform electric field, the corona charge can be deduced from the following steps.

First, the droplet being uncharged is considered, and the potential at *P* point is *Φ*. The result is shown as follow,

14 $${\Phi }=\frac{\mu cos\theta }{4\pi {\varepsilon }_{0}{r}^{2}}-{E}_{0}rcos\theta $$

where *E*_0_ is external electric field, *μ* is the dipole moment, *r* and *θ* represent the distance and angle respectively between the point *P* and centre of droplet.

When point *P* locates on the surface of droplet, it can be obtained as follow,

15 $$\frac{\mu }{4\pi {\varepsilon }_{0}{r}^{2}}-{E}_{0}a=-\,{E}_{t}a$$

where *E*_*t*_ is the inside electric field of droplet, *a* is the radius of droplet. According to the continuity condition of electric displacement,

16 $${\varepsilon }_{0}(\frac{d{\Phi }}{dr})=\varepsilon (\frac{d{{\Phi }}_{t}}{dr})$$

where *Φ*_*t*_ is the potential of droplet, *ε* is the permittivity of droplet. The following equation is derived.

17 $$\frac{\mu }{4\pi {\varepsilon }_{0}}\cdot \frac{2{\varepsilon }_{0}}{{a}^{3}}+{\varepsilon }_{0}{E}_{0}=-\,\varepsilon {E}_{t}$$

According to the Eqs. (16) and (17), the result is shown as follow.

18 $$\mu =(\varepsilon -{\varepsilon }_{0})4\pi {\varepsilon }_{0}{a}^{3}{E}_{0}/(\varepsilon +2{\varepsilon }_{0})$$

Then,

19 $${\Phi }=\frac{\varepsilon -{\varepsilon }_{0}}{\varepsilon +2{\varepsilon }_{0}}{a}^{3}{E}_{0}\frac{cos\theta }{{r}^{2}}-{E}_{0}rcos\theta $$

The electric field *E*_1_ which is produced by droplet on point *P* is:

20 $${E}_{1}=-\,\frac{d\phi }{dr}={E}_{0}cos\theta +2(\frac{\varepsilon -{\varepsilon }_{0}}{\varepsilon +2{\varepsilon }_{0}}){E}_{0}cos\theta \frac{{a}^{3}}{{r}^{3}}$$

Lastly, if the droplet carried with charge *q*, the electric field *E*_2_ on point *P* is:

21 $${E}_{2}=\frac{q}{4\pi {\varepsilon }_{0}\varepsilon {r}^{2}}+{E}_{0}cos\theta +2{E}_{0}(\frac{\varepsilon -{\varepsilon }_{0}}{\varepsilon +2{\varepsilon }_{0}}){E}_{0}cos\theta \frac{{a}^{3}}{{r}^{3}}$$

When *P* locates on surface of droplet and its coordinate is (*a*, π), *E*_2_ = 0, and it means the *q* reaches a maximum value. The maximum *q*_*max*_ is:

22 $${q}_{max}=4\pi {\varepsilon }_{0}{a}^{2}{E}_{0}\frac{3\varepsilon }{\varepsilon +2{\varepsilon }_{0}}$$

The theoretical specific charge is:

23 $${\beta }_{max}=\frac{{q}_{max}}{m}=4\pi {\varepsilon }_{0}{a}^{2}{E}_{0}\frac{3\varepsilon }{\varepsilon +2{\varepsilon }_{0}}/(4\pi \rho {a}^{3}/3)=\frac{{\varepsilon }_{0}{E}_{0}}{\rho a}\frac{9\varepsilon }{\varepsilon +2{\varepsilon }_{0}}$$

where *ρ* is the droplet density, *a* is the droplet radius.

**Figure 8:**
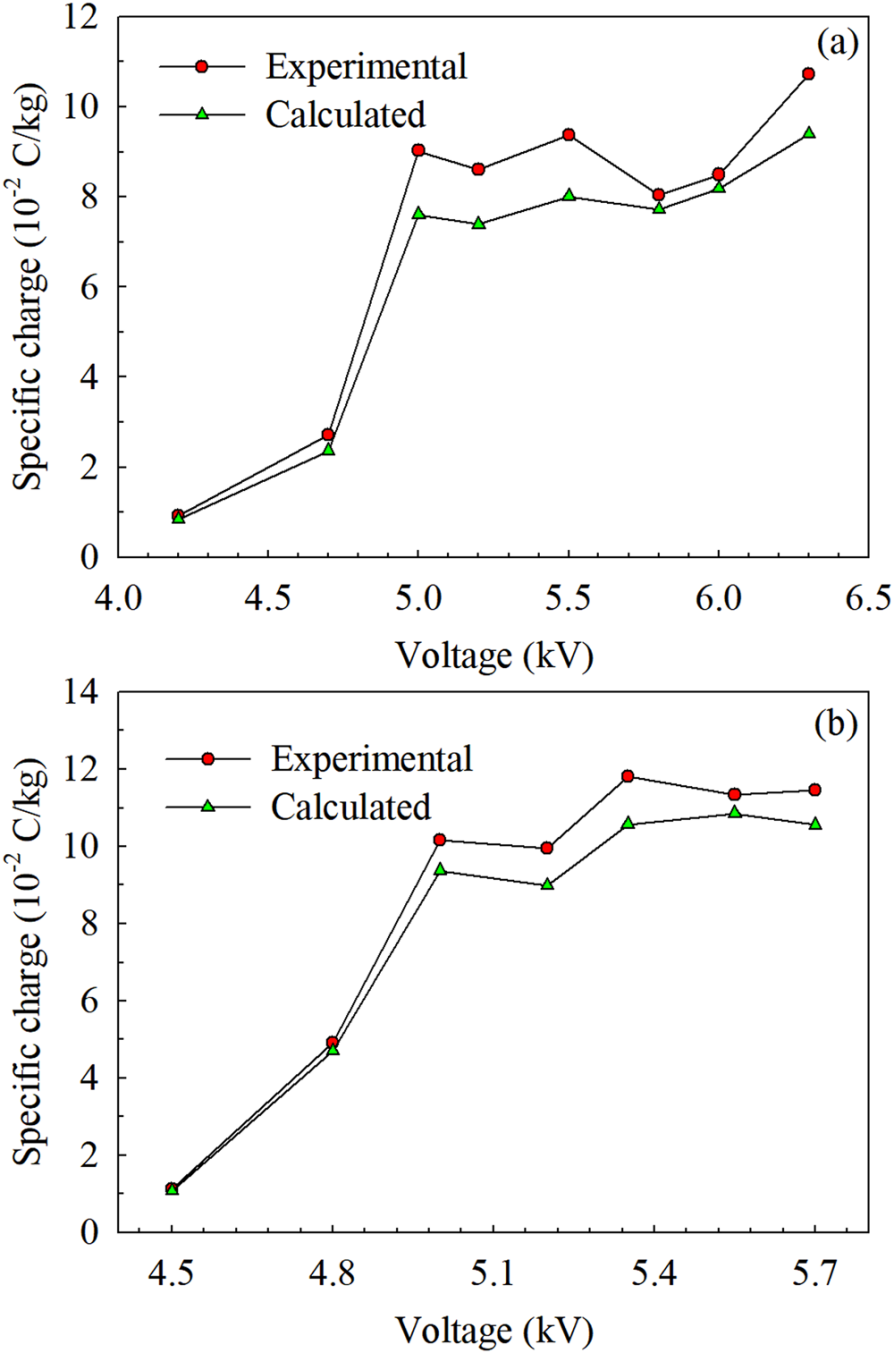
Comparison between calculated specific charge and experimental specific charge. **(a)** Type A: capillary-mesh system; **(b)** Type B: capillary-ring-mesh system (*V*_*r*_ = 1 kV, *L* = 26 mm, *Q*_*v*_ = 1 ml/h).

According to the Eq. (23), the theoretical specific charge is related to the droplet radius *a* and external electric field *E*_0_. The droplet radius *a* is the half of *D*_32_ which measured by the PDA. Meanwhile external electric field *E*_0_ in two electrospray systems can be calculated by the Eqs. (8) and (10). To compared with the experimental results, *a* and *E*_0_ are represented with the mean values of the droplets at the cross-section of z = 10 mm The relation between theoretical specific charge generated by corona charging and experimental specific charge is shown in Fig. 8. It is found that the calculated specific charge generated by corona charging is close to experimental specific charge in two electrospray systems. It can be inferred that the main source of droplet surface charge come from the corona charging process. The result is consistent with that the discharge current is the main part of the total current^38^. The calculated result by the equation of corona charge is in good agreement with the experimental results with the relative error of 11% for Type A and 7% for Type B. And to some extent, the theoretical corona charge can be used to estimate the specific charge in some circumstances.

## Conclusions

A series of comparative experiments were conducted to explore the effects of a ring electrode on the electrospray performance, including the droplet size, droplet velocity and specific charge. To reveal the role of the ring electrode, the electric field of the two different electrospray systems was obtained. With the attempt to theoretically estimate the droplet charge, the corona charge of the droplets was calculated and compared with the experimental results.

Axial electric field intensity in both two electrospray systems reaches the maximum value around the capillary. Meanwhile the axial electric field intensity around the capillary in Type A system is stronger than that in Type B due to the effect of ring electrode. The droplet size in Type B system is smaller than that in Type A system because the ring electrode produces a larger electric force on droplet comparing with Type A and further reduces the droplet size. In the meantime, the axial velocity in Type B system is smaller than that in Type A system in the same spraying mode. The ring electrode plays an important role in uniformity and stability of spraying process.

By measuring the spraying current, the average specific charge of the droplets for the two systems was obtained in different spraying modes. And it was found that the addition of the ring electrode can help to increase the droplet charge, which is the fundamental reason for Type B electrospray system to perform better. The main source of surface charge comes from the corona charging process. The calculated result by the equation of corona charge is in good agreement with the experimental results with the relative error of 11% for Type A and 7% for Type B. It is inferred that the theoretical corona charge can be used to estimate the specific charge of the droplets.
